# Targeting survivin sensitizes cervical cancer cells to radiation treatment

**DOI:** 10.1080/21655979.2020.1717297

**Published:** 2020-01-23

**Authors:** Jing Zhou, Xiaojing Guo, Weifen Chen, Liming Wang, Yonglong Jin

**Affiliations:** a Department of Radiotherapy, The Affiliated Hospital of Qingdao University, Qingdao, Shandong, China; b Reproductive Medicine, The Affiliated Hospital of Qingdao University, Qingdao, Shandong, China; c Gynecology, The Affiliated Hospital of Qingdao University, Qingdao, Shandong, China

**Keywords:** Cervical cancer, radiotherapy, apoptosis, survivin, PUMA

## Abstract

Survivin is an inhibitor of apoptosis protein that functions to inhibit apoptosis, promote proliferation, and enhance invasion. It is selectively up-regulated in many human tumors and implicated in cellular radiation response through its role in apoptosis, cell division, and DNA damage response. This study aimed to investigate the effect and mechanisms of targeting survivin radiosensitivity in cervical cancer C33A cells. Here, the authors designed a small interfering RNA (siRNA) or plasmid-based small hairpin RNA (shRNA) targeting survivin and tested its effects on radiosensitivity to ionizing radiation (IR) treatment of C33A cells *in vitro*, as well as on the tumorigenicity of C33A cells in nude mice *in vivo*. Transient transfection of survivin siRNA into C33A cells suppressed survivin expression, induced cell apoptosis and G2/M arrest and reduced cell proliferation, clone formation ability after IR, followed by p53 upregulated modulator of apoptosis (PUMA) upregulation. But, transient transfection of survivin siRNA alone has no significant effect on cell growth and apoptosis. To confirm that PUMA upregulation is necessary for survivin silencing -induced radiosensitivity to IR treatment, the effect of targeting PUMA in survivin sliencing cells was observed. The results showed that targeting PUMA in survivin sliencing cells rescued C33A cells’ radioresistance. Furthermore, knocking down survivin expression combined with IR treatment significantly slowed tumor growth and promoted tumor cell apoptosis in C33A xenografted tumors. It was concluded that survivin played a role in radiotherapy resistance. Targeting survivin increased the radiosensitivity of C33A cells through induction of PUMA expression.

## Graphical Abstract

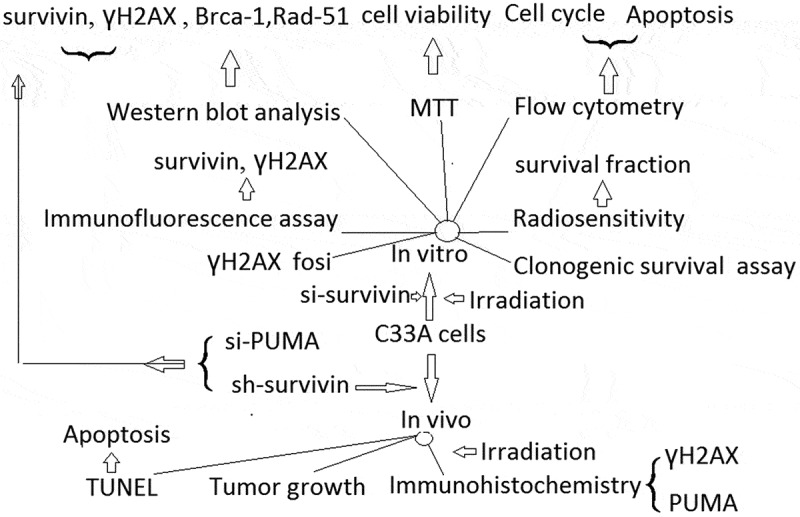

## Introduction

Radiotherapy (RT) has a significant role in definitive and adjuvant therapy for cervical cancer. Investigations showed that RT is used to treat more than 60% of cervical cancer cases [1]. Unfortunately, studies also indicated that the overall incidence of local recurrence is 13% following definitive RT, which suggesting that recurrence after RT remains a problem in the treatment of cervical cancer [2]. The major obstacle to the treatment success of radiotherapy is radioresistance. Moreover, salvaging previously radioresistant tumors using either radiotherapy or surgery with concern for normal tissue complications is difficult. Clinically, the identification and treatment of radioresistant cervical cancer remains an unsolved problem [3]. It is imperative to understand the molecular mechanisms involved in resistance to RT and to use this information to develop novel strategies to enhance the efficacy of RT in cervical cancer patients.

Survivin, belonging to the inhibitor of apoptosis (IAP) family, is a widely expressed anti-apoptotic protein; it participates in regulating the development and progression of many tumors because of its capacity to facilitate cell survival, promote cell cycle progression, and enhance aggressive tumor behavior [4–6]. There is also evidence that survivin suppresses radiation-induced cytotoxicity. In experiments using pancreatic cancer cell lines, survivin mRNA expression and radiosensitivity showed an inverse relationship [7]. In the same set of experiments, survivin expression was increased upon treatment with a sublethal dose of X-irradiation. This inverse relationship between survivin expression and radiosensitivity has also been observed in esophageal squamous cell carcinoma [8], glioma [9], rectal Cancer [10] and prostate cancer cells [11]. In addition, inhibition of the survivin often sensitizes radio-resistant tumor cells in various cancers to irradiation [11–14]. Taken together, these findings suggest that the inhibition of survivin has the potential to also enhance the effects of radiotherapy in cancer patients.

In the present study, we investigated the association between survivin expression and radiosensitivity in cervical cancer C33A cells. Our data shows that survivin overexpression is resistant to radiotherapy, and that this radio-resistant C33A cells is mediated by survivin-PUMA signaling. This suggests that targeting survivin-PUMA signaling might be an effective strategy for adjuvant radiotherapy in the survivin overexpressing cervical cancer.

## Materials and methods

### Cell lines and culture

C33A cells were obtained from American Type Culture Collection (Manassas, VA, USA). It was cultured in Dulbecco’s modified Eagle medium (DMEM) containing L-glutamine, 10% fetal bovine serum (FBS), 1% Non-essential amino acids, and 1% sodium pyruvate.

### Lentiviral shRNA production and infection

The lentivirus expressing the shRNA against scrambled (control) or survivin (sh-survivin) was produced as follows: Lentiviral pLKO.1-puro vectors encoding survivin -specific and scrambled shRNA were purchased from Sigma TRC shRNA library. For lentivirus production, 293T cells were transfected with pLKO.1 vector along with packaging plasmids encoding Gag/Pol, Rev, and VSV-G using the Lipofectamine 2000 reagent (Invitrogen) according to the manufacturer’s instructions. Culture growth media containing lentiviral particles were collected 48 h after transfection and filtered. Viral supernatants were pooled and stored at −80°C. C33A cells were infected with viruses in medium and selected for stable expression of shRNA by treating with puromycin (10 μg/mL) for 2 weeks.

### Transient siRNA transfection

Synthetic siRNA for survivin (si-survivin) and the nonspecific control (si-control) were purchased from Santa Cruz Biotechnology. si-survivin has the sequence 5′-CGUGGUGUUGGUUCGUGAAdTdT-3′ (sense) and 5′-UUCCAGAACACACACAACGdTdT (antisense). As a control, we used single non-targeting siRNAs. C33A cells were seeded (150,000 cells/well) on six-well culture dishes to 50–60% confluence. Then the complete culture medium was replaced with serum-free and antibiotic-free medium at 1 hour before transfection. The cells were incubated with transfection mixtures containing 100 nmol of si-survivin or si-control using the Lipofectamine 2000 according to the manufacturers’ instructions for 5 hours, and then the medium was replaced with full culture medium. After 24–72 hours further incubation, cells at approximately 90% confluence were harvested for further analysis.

To study the effect of PUMA on targeting survivin-induced radiosensitivity to IR treatment of C33A cells, the stably sh-survivin transfected C33A cells were transiently transfected in to si-PUMA for 24 h as the methods above.

### X-ray irradiation

Irradiation of the cells at 65–75% confluence in T75 cell culture flasks (app. 8 × 10^6^ cells) with X-rays was performed using an Xstrahl 320 kV generator (250 kV, 12 mA, 3.8 mm Al, and 1.4 mm Cu) from a medical linear accelerator (Precise accelerator, Elekta, Sweden) at room temperature. Cell culture flasks were horizontally irradiated with different X-rays doses [0, 2, 4, 6, 8 Gy] at a calculated dose rate of 0.5 Gy/min. Field size for the X-ray irradiation was 20 × 20 cm to fully cover the flasks and the source to surface distance was 50 cm. A filter unit comprised of 0.75 mm tin, 0.25 mm copper and 1.5 mm aluminum (half value layer ≈3.7 mm Cu) was used. After treatment, all cell culture flasks were returned to the incubator for 2 h at 37°C and 5% CO2. Control cells were sham irradiated and handled in the same way as irradiated cells. Finally, cells were washed twice with chilled PBS and harvested with a cell scrapper prior to cell lysis.

### Cell viability assay

Exponentially growing cells were transfected with si-survivin/or si-control for 24 h. After the treatments, cells were irradiated at ambient temperature with 2,4, 6, 8 Gy of x-ray (250 keV). Cells were seeded into 96-well plates at 3 × 10^3^ cells per well and allowed to adhere overnight. Cell viability was determined after 72 h using the MTT assay. Plates were read with a SpectraMax 190 microplate spectrophotometer (Beckman Coulter, Inc.) at a wavelength of 540/570 nm.

### Clonogenic formation assay

Exponentially growing cells were transfected with si-survivin/or nonspecific RNA for 24 h. After the treatments, cells were irradiated at ambient temperature with 2 Gy of x-ray (250 keV) and replated into 100-mm-diameter culture dishes at densities calculated to yield 50 to 100 cell colonies per dish. After 10 to 14 days of incubation, cells were fixed and stained with crystal violet in 20% ethanol, and colonies more than 50 cells were counted. The number of Survivin colonies divided by the number of plated cells was used to calculate the plating efficiency and survival fraction for each treatment.

### Flow cytometry analysis

Exponentially growing cells were transfected with si-survivin/or si-control for 48 h. Then the cells were seeded at a concentration of 2 × 10^5^ cells/well in 6-well plates. After incubation for 24 h, the cells were exposed to 2 Gy X-rays. After 24 h, an FITC Annexin V Apoptosis Detection Kit (BD Biosciences, Oxford, UK) was used to detect the apoptotic cells and PI/RNase Staining Buffer (BD Biosciences) was used to detect cell cycle by flow cytometry.

### Immunofluorescence assay

Cells were seeded a concentration of 5 × 10^4^ into confocal laser small dishes and harvested at 2 hours post IR. Cells were subsequently fixed in 4% paraformaldehyde at room temperature for 30 minutes and permeabilized in 0.1% Triton X-100 (Sigma, Santa Clara, CA, USA) for 15 minutes. The cells were then blocked with 5% BSA (Gibco, NY, USA) for 1 hour and incubated with primary antibody γH2AX (1 μg/mL; Abcam, Cambridge, Cambridgeshire, UK) overnight at 4°C. Cells were washed in Tris-Buffered Saline Tween-20 (TBST) 3 times every 5 minutes before incubating with a secondary Ab (Beyotime Biotechnology) for 1 hour. Cells were treated with 2 μg/mL DAPI (Beyotime Biotechnology) for 5 minutes and then visualized using confocal fluorescence microscopy (Leica, Frankfurt, Germany).

### Western blot analysis

Equal amounts of total protein from conditioned media or cell lysates obtained by lysing cells in a suitable buffer [50 mmol/L Tris-HCl (pH 8.0), 150 mmol/L NaCl, 1% NP40, 0.5% sodium deoxycholate, 0.1% SDS, 0.5 mmol/L henylmethylsulfonylfluoride] were separated on second dimensional sodium dodecyl sulfate-polyacrylamide gel electrophoresis (SDS-PAGE) and transferred to polyvinylidene difluoride membranes (Bio-Rad). After blocking with 6% nonfat milk and 0.1% Tween 20 in TBS, membranes were incubated with anti- survivin, γH2AX, PUMA,Brca-1,Rad-51 antibody (Chemicon, Temecula, CA), followed by incubation with anti-mouse secondary antibody. Membranes were developed using the ECL system (Amersham Bioscience, Piscataway, NJ). Glyceraldehyde-3-phosphate Dehydrogenase (GAPDH) antibody was used as a loading control.

### In vivo experiments

Mouse care and experimental procedures were performed under pathogen-free conditions in accordance with established institutional guidance and approved protocols from the institutional animal care and use committees of the Affiliated Hospital of Qingdao University. For subcutaneous (SC) tumor challenge, we injected 5 × 10^6^ of C33A, C33A/sh survivin,C33A/shcontrol tumor cells into immunodeficient 4- to 6-week-old mice. When the tumor size reached 50–100 mm^3^ (day 1), the tumors were irradiated 8 Gy. Mice were killed on day 28 after IR (8 Gy). Tumor volumes were measured every other day. Tumor volumes were calculated by measuring the length [L] and width [W] of tumors using callipers. The formula tumor volume = (L × W2)/2 was used to calculate the tumor volume.

### Immunohistochemistry and TUNEL of tumor tissues

The immunohistochemical analysis was perfomed as the normal method. Briefly, unstained sections of tumor tissues were deparaffinized and rehydrated. Antigen retrieval was performed with DAKO antigen retrieval solution (DAKO, North America Inc., Carpinteria, CA). Endogenous peroxidase was blocked by hydrogen peroxide (3%). For protein blocking, IgG blocking from a Vector M.O.M. kit (Vector Laboratories, Inc., Bulingame, CA) was applied for 1 h (for PUMA) or 5% normal horse serum and 1% normal goat serum in PBS were used (for γH2AX). Primary antibodies against γH2AX and PUMA were incubated overnight at 4°C. Slides were developed with DAB substrate (Vector Labs) and counterstained with Gill’s no. 3 hematoxylin solution. The number of positive (DAB-stained) cells was counted in five random fields per slide (one slide per mouse, 5 slides per group) a 200× magnification.

For TUNEL assay, formalin-fixed tumor tissues harvested 28 days after tumor implantation were embedded in paraffin and sectioned. DeadEnd™ Colorimetric Apoptosis Detection System (Promega, Madison, WI) was used to detect apoptosis in the tumor sections placed on slides according to the manufacturer’s protocol. Briefly, the equilibration buffer was added to slides and incubated for 10 min followed by 10-min incubation in 20 μg/ml proteinase K solution. For the rest of the assay, the steps mentioned in section 2.4 were repeated.

### Statistics

Unless otherwise noted, data were expressed as means ± SEM of at least three determinations. Statistical comparisons between different groups were performed with Student’s *t* test or ANOVA, and *P* ≤ 0.05 was considered significant.

## Results

### *Targeting survivin increases the IR sensitivity* in vitro

**Figure 1.**
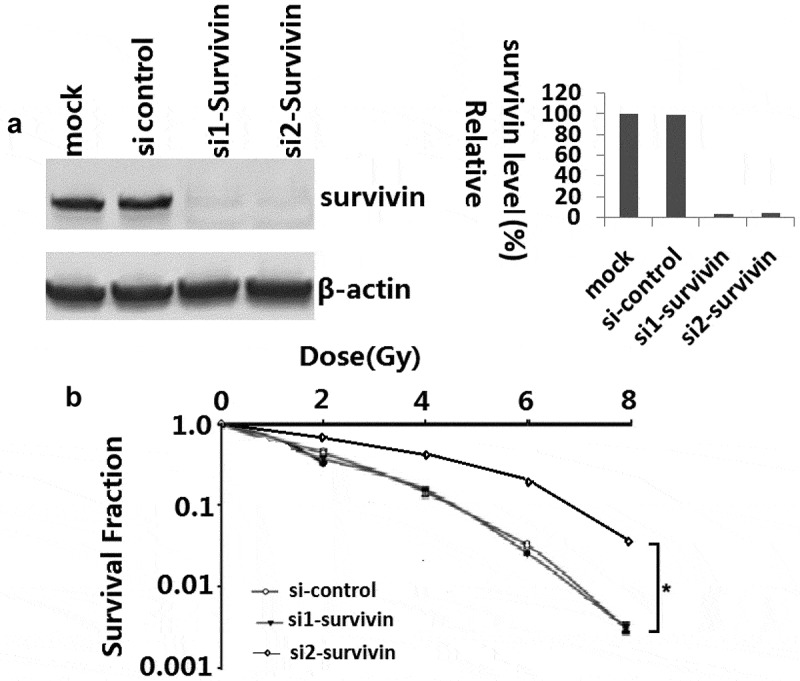
Survivin knockdown resulted in irradiation sensitivity in C33A cells. (a) C33A cells were transfected with scrambled siRNA or si-survivin 1–2 for 72h, and total cell lysates were harvested followed by immunoblotting with the indicated antibodies. (b) Colony formation assays were conducted in C33A cell lines. The differences between si-survivin 1–2 and si-control selected to be compared were detected using repeated measures ANOVA. *, P < 0.05.

In order to examine whether survivin was involved in response to IR, we knocked down survivin gene expression in C33A cells with siRNA oligos (si- survivin*-1* and si-survivin −2) targeting the survivin gene. Western blot analysis showed that survivin was depleted by siRNA (Figure 1(a)). The C33A cells were treated with various doses of IR, and then seeded on cell culture plates for colony formation. Interestingly, C33A cells infected with the si-survivin were more sensitivity to IR than the untreated cells and those treated with control scramble siRNA (Figure 1(b)). These results indicate that suppression of survivin expression decreases radioresistance in C33A cells.

### *Targeting survivin increases IR-induced DNA damage and genomic instability* in vitro

**Figure 2.**
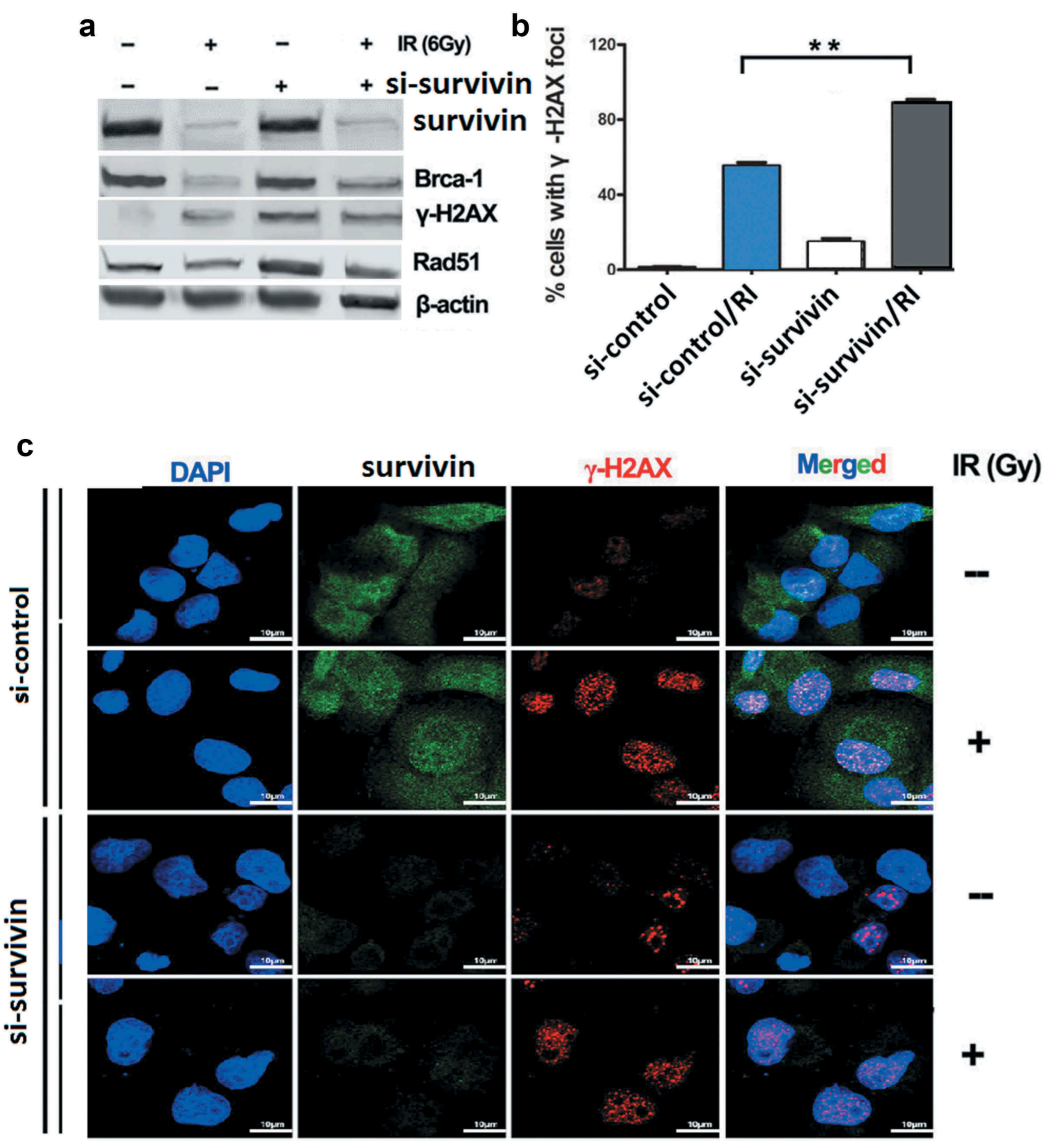
*Targeting* survivin impaired DNA Double-Strand Break Signaling in C33A cells in response to IR. (a) C33A cells were transfected with scrambled siRNA, or si-survivin for 72 h, and then total cell lysates were harvested followed by immunoblotting with the indicated antibodies. (b, c) C33A cells were transfected with scrambled siRNA or si-SURVIVIN and then exposed to IR (6 Gy). Laser confocal microscopy images of C33A labeled with fluorescent antibodies to γ-H2AX, survivin and DAPI at 2 h post irradiation or mock treatment. Focal g-H2AX staining signals were quantified in cells. (c) The typical staining of γ-H2AX inC33A cells before and after the treatment with irradiation. The average data point was calculated from three independent experiments. At least 1000 cells were counted for each cell line. The differences in those two groups were detected using t-test. **, *P* < 0.01.

Radiation kills cancer cells by inducing DNA damage which triggers DNA damage response (DDR), including genomic instability. To investigate whether targeting survivin affects genomic stability, we examined several DDR endpoints. Targeting survivin activated phosphorylation of the H2AX histone (γ-H2AX), a molecular marker for DNA double strand breaks (DSB), in C33A cells without IR (Figure 2(a)). After IR treatment, the γ-H2AX level increased in both control and survivin depleted cells (Figure 2(b)). Interestingly, the levels of Brca1 and Rad51, two key proteins involved in DNA DSB repair, were reduced in cells with suppressed survivin expression (Figure 2(a)). There was a larger number of γ-H2AX foci in C33A cells transfected with si-survivin after IR treatment, and massive staining of γ-H2AX foci were readily visible within 2 h of IR in the nuclei of C33A cells treated with either the control siRNA or si-survivin (Figure 2(b,c)). These results indicate that the survivin protein is required for maintaining a proper DNA damage response machinery in cancer cells.

### *Targeting survivin increases IR-induced apoptosis and prolongs IR-induced G2/M arrest* in vitro

**Figure 3.**
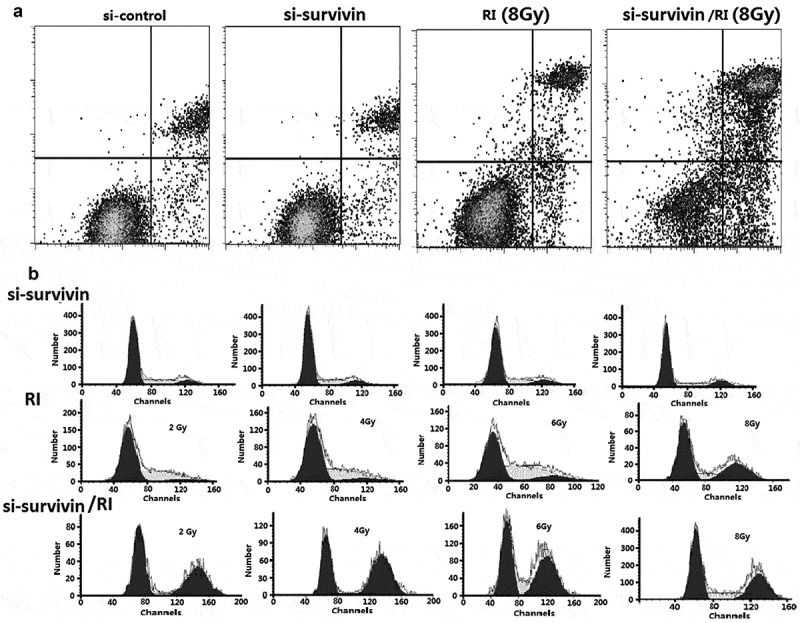
Targeting survivin IR-induced apoptosis and prolongs IR-induced G2/M arrest. (a) Flow cytometry was used to analyze apoptosis in C33A cells after si- survivin transfection or/and 6 Gy IR for 24 hours. (b) Cell cycle was detected by flow cytometry after 2–8 Gy IR after 24 hours.

To further illustrate the role of silencing survivin and IR (6Gy) in apoptosis, we evaluated apoptosis by flow cytometry. The total apoptosis rate, including early and late apoptosis populations, was calculated. In our research, IR alone-induced apoptosis did not increase significantly; however, si-survivin combined with IR can significantly increase the rate of apoptosis in C33A cells (Figure 3(a)).

Sensitivity to IR is different in different cell cycles. The G2/M phase is the most radiosensitive. We used flow cytometry analysis to clarify the cell cycle progression. We found that IR blocked cells in the G2/M phase after 24 hours and the result was the same as si-survivin alone (Figure 3(b)). Si-survivin combined with IR could significantly increase the proportion of the G2/M period compared with single treatment (Figure 3(b)). Therefore, we could have inferred that silencing survivin increased radiosensitivity by inducing G2/M arrest.

### *Targeting survivin upregulates PUMA expression* in vitro *after irradiation exposure*

**Figure 4.**
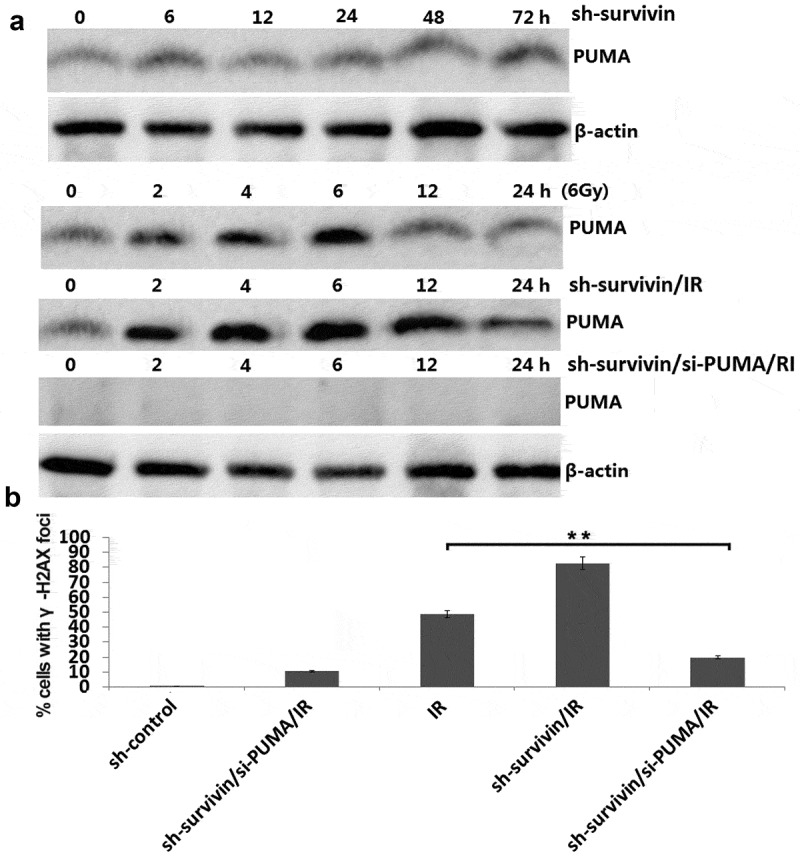
Survivin depletion induced high level of PUMA in response to IR. (a) C33A cells were transfected with sh-survivin or/and si-PUMA and then were treated with or without IR (6 Gy). Cell lysates were harvested at the indicated time points after IR. (b) Focal g-H2AX staining signals were quantified in cells. **P < 0.01.

We next examined PUMA levels in survivin -depleted C33A cells. PUMA levels were increased within 2 to 6 hours after IR (6Gy) treatment in C33A cell lines (Figure 4(a)). The IR-induced PUMA levels were higher in survivin -depleted cells than those in control cells within 2 to 6 hours after IR treatment (Figure 4(a)).Targeting survivin alone did not affect PUMA expression in the C33A cells (Figure 4(a)).

### *Targeting PUMA reverse survivin -depletion induced IR sensitivity* in vitro

Since survivin depletion caused higher level of PUMA production in C33A cells after IR (6Gy) treatment, we set up experiments to test whether PUMA play a critical role on IR sensitivity induced by survivin depletion in C33A cells. We transfected si-PUMA in the shRNA or sh- survivin transfected C33A cells for 24 h, then subjected to 6Gy of IR treatment, and then seeded for colony formation. The results showed that PUMA was completed inhibited in the sh- survivin/si-PUMA/IR treated C33A cells (Figure 4(a)). There was a few number of γ-H2AX foci in C33A cells treated with sh- survivin/si-PUMA after IR treatment compared to the C33A cells treated with sh- survivin after IR treatment (Figure 4(b)). These results indicate that the PUMA upregulation is required for SURVIVIN silencing induced IR sensitivity.

### *Targeting survivin increases the radiosensitivity to IR* in vivo

**Figure 5.**
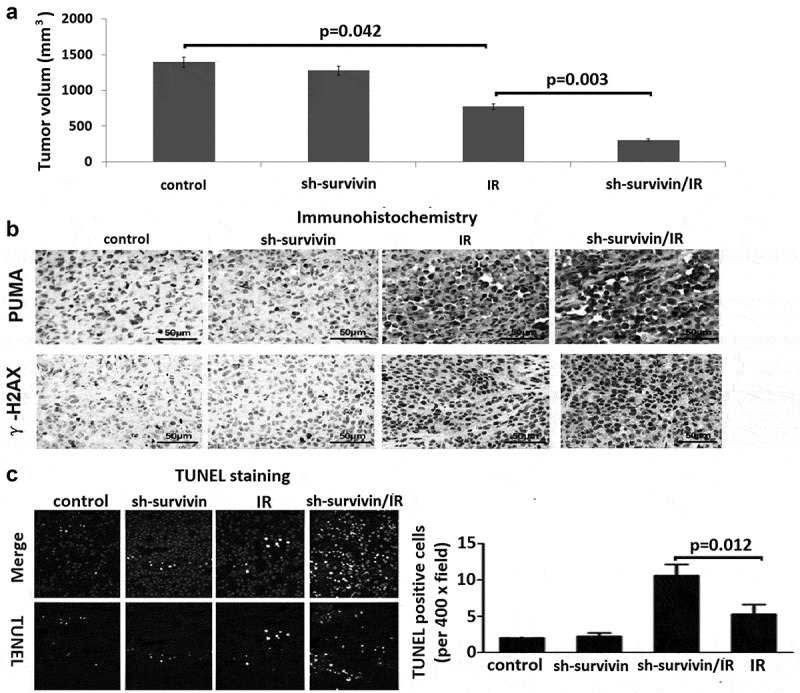
Targeting survivin induced radio-resistance in cervical tumor mouse model. (a) Nude mice were injected with 1 × 10^6^ C33A cells infected with control retroviral vector, or sh-survivin vector with 5% Matrigel into the right back. When the tumor size reached 50–100 mm^3^ (day 1), the tumors were irradiated 8 Gy dose of IR every other day. The mice were sacrificed at day 28, and the tumors were isolated, the **volum**e was compared between the groups using ANOVA. (b) Immunohistochemistry (IHC) was performed on serial sections using antibodies against PUMA and g-H2AX in tumor tissues. (c) Cell apoptosis was detected by TUNEL staining.

To validate whether the above cell-based findings were applicable *in vivo*, we generated subcutaneous C33A xenografts in the right posterior flanks and treated tumor-bearing mice with IR (Figure 5(a)). Growth of the control C33A tumors was significantly inhibited by IR treatment (P = 0.042), survivin depletion largely promoted radio-sensitivity of C33A tumors (P = 0.003). We found that γ-H2AX and PUMA expression level was less increase in tumor sections derived from sh-survivin expressing cells than those from sh-control cells, but γ-H2AX and PUMA expression levels were further increased after IR (Figure 5(b)). In addition, apoptotic cells were significantly increased in sh-survivin expressing xenografts by IR treatment (Figure 5(c), P = 0.012). These results suggest that survivin is essential for cervical cancer radiosensitivity.

## Discussion

Radioresistance is mainly responsible for treatment failure and mortality in cervical cancer patients who receive radical radiation therapy [15]. However, the mechanisms underlying this resistance are only partially defined. Therefore, there is an urgent need to discover new markers and therapeutic targets to overcome this problem.

Survivin is a unique member of the IAP family that is expressed in most human tumors, but is barely detected in normal adult tissues [16,17]. Overexpression of survivin in tumors is generally associated with poor prognosis radioresistance and drug resistance [18–21], the underlying mechanisms are not fully understood. Previous studies suggested that survivin enhances the survival of tumor cells primarily through the suppression of apoptosis by inhibition of caspases [22]. Recent studies have suggested a link between survivin and radiation-induced DNA damage response. Accordingly, interference with survivin expression or function resulted in reduced DNA repair [23,24]. In this study, we found that targeting survivin increased the radiosensitivity and promoted the apoptosis of C33A cells both *in vitro* and *in vivo*. Combined survivin sliencing and radiation treatment increased PUMA expression levels, impairing the irradiation-induced G_2_/M arrest. Targeting survivin decreased the G2/M arrest induced by radiation, which most likely resulted in more irradiation-damaged cells entering mitosis. Moreover, the combined treatment of survivin sliencing and irradiation increased apoptosis in cultured C33A cells and C33A xenografts

The phosphorylated form of H2AX (γH2AX) is recognized as a histone involved in the event of DNA damage, and there is a one-to-one relationship between the numbers of γH2AX foci with DNA breaks caused by IR. γH2AX has been well used as a marker of DNA double-strand breaks [24]. In the present study, we found that silencing survivin increased IR-induced DNA damage and reduced DNA repair. Radiation-induced arrest at G2-M is critical in preventing cell death. This study shows that in combination with radiation, survivin sliencing causes a greater number of cells to be arrested at G2-M, which implies a larger proportion of cancer cells are disrupted by combination therapy. Using a xenograft nude mouse model, we showed that silencing survivin with IR enhanced the radiosensitivity of C33A cells in vivo. The combination of survivin silencing and IR notably suppressed tumor volume compared with the other treatment groups.

The p53 upregulated modulator of apoptosis (PUMA) is a member of the ‘BH3-only’ branch of the Bcl-2 protein family, members of which are shown to initiate apoptosis in a tissue- and stimulus-specific manner. Yu et al. has found that the BH3-only protein p53 upregulated modulator of apoptosis (PUMA) deficiency had little effect on radiation-induced intestinal endothelial apoptosis, and suppressing PUMA expression by antisense oligonucleotides provided significant intestinal radioprotection [25]. Shao et al. has reported that PUMA deficiency in mice confers resistance to high-dose radiation in a hematopoietic cell-autonomous manner. Unexpectedly, loss of one PUMA allele is sufficient to confer mice radioresistance [26]. Yan et al. has reported that targeting survivin by YM155 induced apoptosis by activating PUMA-caspase-3 pathway in human oral squamous cell carcinoma (OSCC) cells [27].

In the study, targeting survivin upregulates PUMA in C33A cells after IR exposure and PUMA upregulation is required for survivin silencing-induced IR sensitivity. In vivo, silencing survivin with IR induced significant cell apoptosis, followed by increased PUMA and γH2AX expression.

## Conclusions

In conclusion, this study showed that targeting survivin enhanced the radiosensitivity to IR in C33A cells *in vitro* and *in vivo*. The mechanisms of radiosensitization might be aggravating DNA damage, weakening DNA repair, and inducing G2/M arrest and apoptosis. PUMA is a potential molecular target of radiosensitization. The potential radiosensitizing effects of targeting survivin on C33A cells suggested that survivin may be an effective target for treatment of cervical cancer.

## Research highlights

1. Targeting survivin alone has no significant effect on C33A cell growth and apoptosis *in vitro* and *in vivo*.
2. Targeting survivin inhibited IR-induced PUMA upregulation in C33A cells *in vitro* and *in vivo*.
3. Targeting survivin increased the IR sensitivity of C33A cells via PUMA upregulation *in vitro* and *in vivo*.
